# Emotion-Specific Affective Theory of Mind Impairment in Parkinson’s Disease

**DOI:** 10.1038/s41598-018-33988-6

**Published:** 2018-10-30

**Authors:** Rwei-Ling Yu, Po See Chen, Shao-Ching Tu, Wei-Chia Tsao, Chun-Hsiang Tan

**Affiliations:** 1 Institute of Behavioral Medicine, College of Medicine, National Cheng Kung University, Tainan, Taiwan; 2 Department of Psychiatry, National Cheng Kung University Hospital, College of Medicine, National Cheng Kung University, Tainan, Taiwan; 3 Institute of Allied Health Sciences, College of Medicine, National Cheng Kung University, Tainan, Taiwan; 4 Department of Neurology, National Taiwan University Hospital, College of Medicine, National Taiwan University, Taipei, Taiwan; 5 School of Medicine, College of Medicine, Kaohsiung Medical University, Kaohsiung, Taiwan; 6 Department of Neurology, Kaohsiung Medical University Hospital, Kaohsiung Medical University, Kaohsiung, Taiwan; 7 Graduate Institute of Clinical Medicine, College of Medicine, Kaohsiung Medical University, Kaohsiung, Taiwan

## Abstract

The neuropathology of Parkinson’s disease (PD) involves the frontal-subcortical circuit, an area responsible for processing affective theory of mind (ToM). Patients with PD are expected to experience deficits in the affective ToM. This study aims to investigate whether the ability to infer emotion in others is affected in either young-onset Parkinson’s disease (YOPD) or middle-onset PD (MOPD) patients and to test whether the impairments in affective ToM are associated with the motor symptoms. The affective ToM, global mental abilities, and clinical symptoms were assessed in a total of 107 MOPD, 30 YOPD, and 30 normal controls (NCs). The MOPD patients exhibited deficits in affective ToM to the negative and neutral valences, when compared to the participants in the NCs and YOPD group. By conducting gender-stratified analysis, the deficits in affective ToM was only found in female participants. After adjusting for demographic variables, the multiple linear regression model revealed that affective ToM predicted motor symptoms, especially in female MOPD patients. The present study may aid in the development of medical care programs by advocating for a more comprehensive therapeutic plan that includes continuous disease progression monitoring and social skills training for female MOPD patients or their caregivers.

## Introduction

With advances in the understanding of the neuropathological stages of Parkinson’s disease (PD), the latest research and treatment have focused on the non-motor symptoms of the disease, such as executive function^1^. Emerging evidence supports that non-motor symptoms (e.g., cognitive dysfunction, loss of smell, constipation) influence not only the disease progression but also the quality of life^2^. The non-motor symptoms may even occur before the motor symptoms; therefore, recent discussion of the definition of PD diagnosis has shifted from the original motor symptoms to non-motor symptoms^3^. The latest edition of the American Psychiatric Association’s Diagnostic and Statistical Manual of Mental Disorders introduced social cognition as one of the six core components of neurocognitive function, alongside memory and executive function^4^. Social cognition broadly refers to the processing of social information in the brain that underlies abilities such as the detection of others’ emotions and responding appropriately to these emotions. The abilities to interact effectively in a social situation and to infer other’s emotions correctly are fundamental skills for successful communication^5^.

A key component in the ability to infer other’s feelings involves an intact affective theory of mind (ToM)^6^. ToM is fundamental in everyday life but is a relatively new area in PD research^7^. The RMET is a traditional and well-known assessment of the affective ToM^8^. The test presents the participants with a series of photographed pairs of eyes that each express a given emotion and asks the participants to pick an adjective that best fit the emotion expressed by the photograph. While the effects of PD on cognitive ToM have been consistent^9^, current literature has yet to reach a consensus on the manifestation of PD and its effect on the patient’s affective ToM functions. Considering that the ventromedial prefrontal regions and the orbital frontostriatal circuit are crucial for processing affective ToM^10,11^ and that the neuropathology of PD involves the frontal-subcortical circuit^12^, PD patients are expected to experience difficulty in decoding others’ emotions. Based on such pathology, we assume that affective ToM is impaired in a progressive manner during the course of PD, and the affective ToM may be closely associated with motor symptoms.

Previous studies on the subject have yielded varying results. These controversial results might have been due to the heterogeneity of the PD population and methodological differences employed by the studies. For example, the patients selected in the studies by Peron *et al*.^13^ and Enrici *et al*.^14^ were relatively young (average age: 56 and 57 years old, respectively); taking into account the disease duration (10.2 and 10.5 years), the PD patients represented in these studies were patients with young-onset PD (YOPD) rather middle-onset PD (MOPD), the more representative group for PD^15^. A few studies have investigated the abilities of emotion detection in YOPD patients. Yoshimura and colleagues investigated the ability to recognize facial expression in a small PD sample (n = 16), and they found the emotion state decoding ability in YOPD was preserved. This intact ability may be attributed to the pathophysiology of YOPD, in which less involvement of the mesocorticolimbic system^16^ is expected. Gender differences in emotion recognition have almost been neglected in the PD field^17^. Few studies have focused on this topic. One study found that male PD patients had poor emotional recognition ability in a small PD population (10 patients in each of the male and female groups)^18^. Another study found that male but not female PD patients exhibited confined impairment in recognizing anger emotions^19^. The profile of affected ToM in different genders is not yet clear.

Another possible explanation for the inconsistent results is the various versions of the RMET used by those studies. The number of items used may explain the conflicting results^20^. The RMET versions used in some studies have been abbreviated versions in which 15^21^, 17^13,22^, 20^23^, and 24^24^ stimuli were used instead of the usual 36^14,25–30^, and the patients were asked to choose between 2 instead of 4 adjectives^21,22^. Furthermore, the sample sizes in previous studies had the tendency to be small^13,21,23–29^, with only a few studies recruiting more than 30 patients per group^14,22^. Likewise, the small sample sizes may have failed to reflect a complete clinical picture for the majority of PD patients. Moreover, the emotions depicted in the RMET can further be divided into three valences, positive, negative, and neutral^31^. While previous studies had only compared the RMET total scores between PD patients and those in the control group^13,14,21,23,25,27,29^, the present study was also interested in the differences in the subscores between the three valences, which could provide more data on the perception of the individual emotional valences.

The primary aim of this study was to investigate whether the ability to interpret emotion from a pair of photographed eyes was affected in patients with PD (either YOPD or MOPD patients); and if so, for which specific emotional valences (positive, negative, or neutral) was affective ToM hindered and for which did it remain intact. The second purpose of this study was to test the hypothesis that the motor symptoms are related to affective ToM. Furthermore, we explored the gender differences in the above two goals.

## Results

The demographic and clinical data are summarized in Table 1. There were no significant differences between the gender, educational level, or mental state of the 3 groups. There were no significant differences in the disease duration, Hoehn and Yahr (H&Y) stage, levodopa equivalent daily dose (LED), motor function (Motor Examination, MEx), motor experiences of daily living (Motor Experiences of Daily Living, motor-EDL), or non-motor experiences of daily living (Non-motor Experiences of Daily Living, nM-EDL) of the Movement Disorder Society – Unified Parkinson’s Disease Rating Scale (MDS-UPDRS) between the 2 patient groups. The mean age of onset of the YOPD group was reasonably lower than that of the MOPD group. In addition, the mean age of the MOPD group and normal controls (NCs) were comparable, and the YOPD group was significantly younger than the other two groups.

**Table 1:** Demographic Characteristics and Mental State of the Study Groups.

	NCs (n = 30)			YOPD (n = 30)			MOPD (n = 30)			Stat. Value	P-value	Post Hoc
	Mean	SD	Range	Mean	SD	Range	Mean	SD	Range			
Gender, F/M	19/11	—	—	13/17	—	—	14/16	—	—	2.75^a^	0.252	n.s.
Education, y	12.05	4.14	3–18	12.76	3.13	6–16	12.40	3.36	6–16	0.412^c^	0.814	n.s.
Age, y	58.66	7.12	51–75	53.22	5.29	41–63	61.40	5.43	54–75	25.104^c^	<0.001	YOPD < NCs = MOPD
Age of onset, y	—	—	—	45.01	3.97	36–49	54.93	3.83	50–63	<0.001^b^	<0.001	YOPD < MOPD
MMSE	28.20	1.66	24–30	29.06	1.14	25–30	28.56	1.27	25–30	5.645^c^	0.059	n.s.
Disease duration, y	—	—	—	8.13	4.58	0.66–17	6.43	3.79	2–16	348.50^b^	0.132	n.s.
H&Y stage	—	—	—	2.30	0.95	1–4	2.30	0.83	1–4	445.50^b^	0.944	n.s.
LED	—	—	—	918.26	522.04	120–2196	699.87	438.01	200–1830	332.00^b^	0.081	n.s.
MDS-UPDRS												
nM-EDL	—	—	—	9.03	6.48	0–29	8.64	5.23	1–24	384.50^b^	0.902	n.s.
motor-EDL	—	—	—	12.46	8.70	0–29	10.86	7.32	0–30	376.50^b^	0.637	n.s.
MEx	—	—	—	26.50	12.50	5–46	30.32	15.03	7–65	0.789^d^	0.306	n.s.

Abbreviations: NCs, normal controls; YOPD, young-onset Parkinson’s disease; MOPD, middle-onset Parkinson’s disease; SD, standard deviation; n.s., not significant; H&Y, Hoehn and Yahr; MDS-UPDRS, Movement Disorder Society–Unified Parkinson’s Disease Rating Scale; nM-EDL, non-motor experiences of daily living; motor-EDL, motor experiences of daily living; MEx, motor function; LED, levodopa equivalent dose; MMSE, Mini-Mental State Examination.

^a^chi-square test; ^b^Mann-Whitney U test; ^c^Kruskal-Wallis test; ^d^T-test.

**Figure 1:**
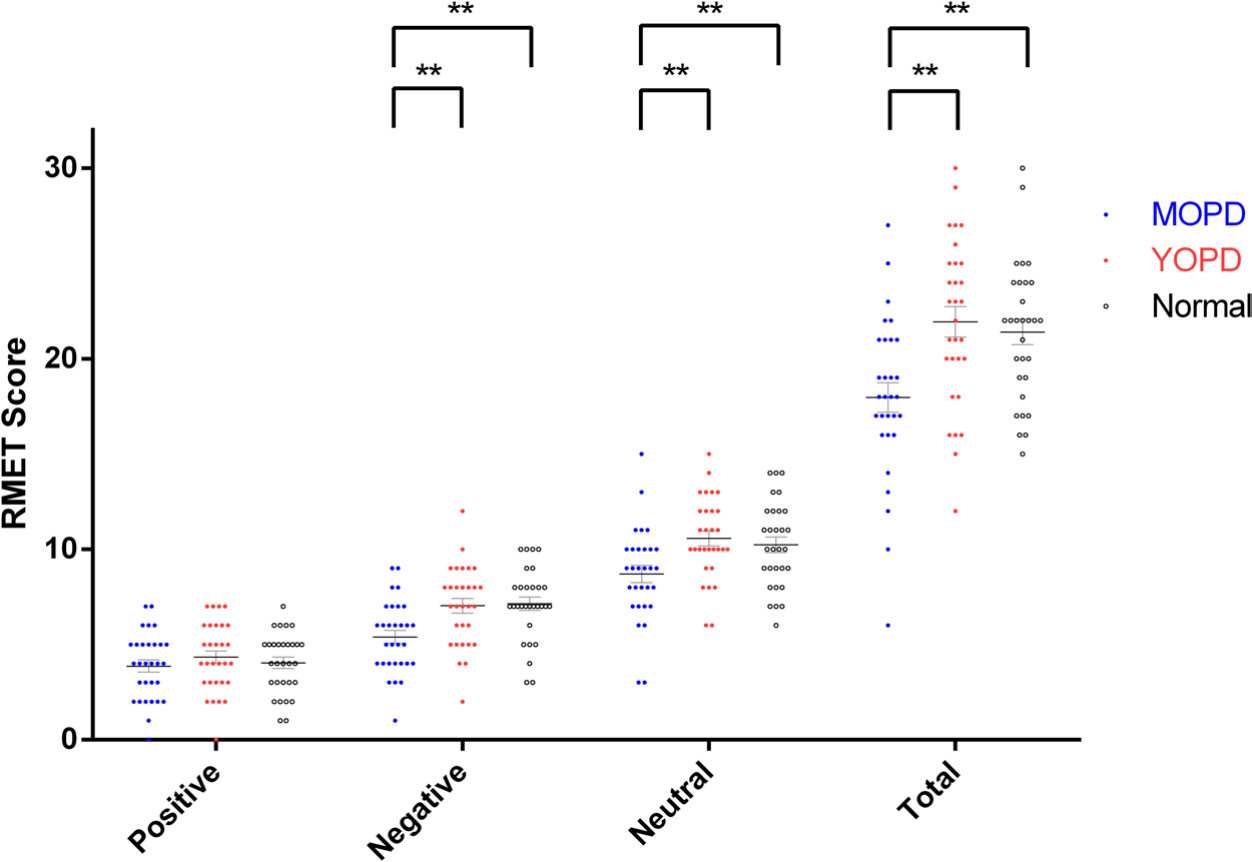
The distribution of the RMET scores in the study groups (Normal = 30, YOPD = 30, MOPD = 30). Mean values and standard error of the mean (SEM, vertical bars) of RMET total score and three valence (e.g., positive, negative, neutral) scores in the three study groups. YOPD, young-onset Parkinson’s disease; MOPD, middle-onset Parkinson’s disease. *P-value < 0.05. **P-value < 0.01.

As shown in Fig. 1, the ANOVA revealed a significant main effect of group in the total score of RMET (F_2,87_ = 8.217, *p* = 0.001). Post hoc tests indicated that the MOPD group had the worst performance on the RMET. Out of the three emotional valences of the RMET (positive, negative, and neutral), the MOPD group performed significantly poorly on decoding negative (Kruskal-Wallis chi-squared = 13.553, df = 2, p = 0.001) and neutral valences (F_2,87_ = 5.660, *p* = 0.001) compared to the YOPD or the NC group. No significant difference was found in the positive valence (F_2,87_ = 0.568, *p* = 0.569) among the three study groups.

In view of the impact of gender on the RMET performance, gender-stratified analysis of RMET was conducted. As shown in Table 2, a significant main effect of group was found in the female population; however, this was not the case for male PD patients.

**Table 2:** Gender-stratified analysis of the RMET.

	NCs			YOPD			MOPD			Stat. Value	P-value	Post Hoc^c^
	Mean	SD	Range	Mean	SD	Range	Mean	SD	Range			
RMET in males	19.45	4.11	15–29	20.88	4.53	12–29	18.81	5.00	6–27	0.870^a^	0.428	n.s.
Positive valence	3.36	1.75	1–6	4.24	2.02	0–7	4.06	2.05	0–7	0.690^a^	0.505	n.s.
Negative valence	6.36	2.34	3–10	6.47	1.84	2–9	5.69	1.82	3–9	1.872^b^	0.392	n.s.
Neutral valence	9.73	2.41	6–14	10.18	2.21	6–14	9.06	3.13	3–15	0.740^a^	0.481	n.s.
RMET in females	22.53	2.93	17–30	23.31	3.90	16–30	17.00	3.16	10–21	15.510^a^	<0.001	NCs = YOPD > MOPD
Positive valence	4.42	1.39	2–7	4.46	1.56	2–7	3.64	1.39	2–6	1.490^a^	0.237	n.s.
Negative valence	7.58	1.46	5–10	7.77	2.24	4–12	5.07	1.98	1–8	9.380^a^	<0.001	NCs = YOPD > MOPD
Neutral valence	10.53	2.14	7–14	11.08	2.02	8–15	8.29	1.44	6–10	8.340^a^	0.001	NCs = YOPD > MOPD

Abbreviations: see Table 1 and RMET, reading the mind in the eye test.

^a^ANOVA; one-way analysis of variance.

^b^Kruskal-Wallis test.

^c^Scheffe post-hoc test.

**Figure 2:**
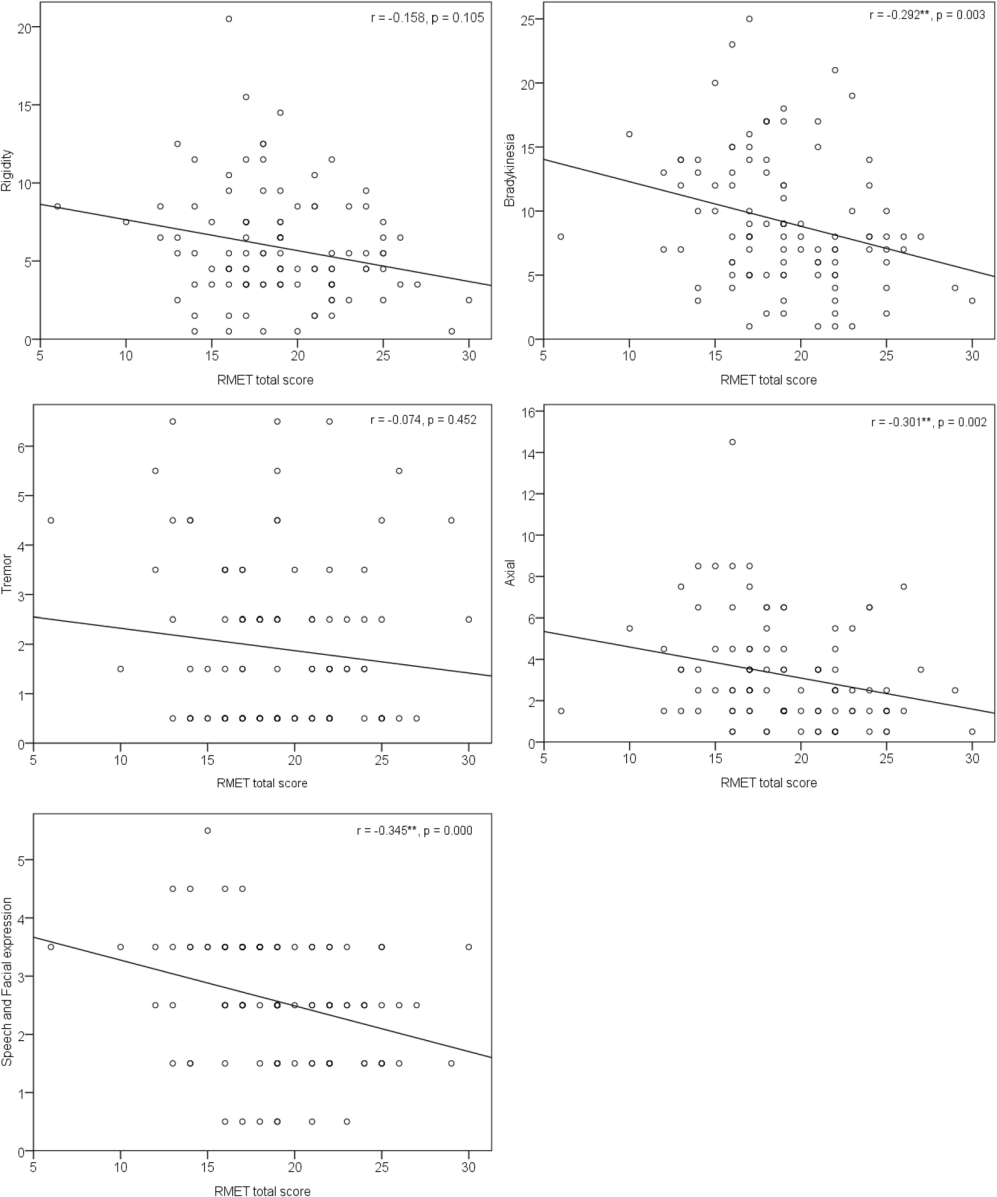
Correlations between the RMET total score and specific motor symptoms.

To obtain a better understanding of the relationship between RMET and PD-related clinical variables (motor and non-motor symptoms) in the 107 MOPD patients in this study, correlation analysis and multiple linear regression were conducted. Regarding the association between the RMET total score and demographic and PD-related variables, the RMET total score was positively correlated with level of education (r = 0.274, p = 0.004) and negatively correlated with prolonged disease duration (r = −0.249, p = 0.010), H&Y stage (r = −0.214, p = 0.027), nM-EDL score (r = −0.339, p = 0.001), motor-EDL score (r = −0.299, p = 0.002), and MEx score (r = −0.321, p = 0.001). No correlation was found between RMET total score and age, onset age, or LED. Concerning the correlation between the RMET total score and the specific motor symptoms (e.g., axial symptoms and bradykinesia), the RMET total score was negatively correlated with the development of bradykinesia (r = −0.292, p = 0.003), axial symptoms (r = −0.301, p = 0.002), and speech and facial expression (SFE) (r = −0.345, p < 0.001) (Fig. 2).

We further performed 12 multiple linear regression models using as dependent variables the motor and non-motor symptoms (e.g., nM-EDL, motor –EDL, and MEx score). Each regression model was adjusted for age, education, gender, disease duration, and LED. Four significant regression equations were found. In the whole PD population, the RMET total score and negative valence score were predictors of the motor-EDL and MEx scores. Because gender has an impact on ToM, we further performed 12 multiple linear regression models in each of the male and female populations. Significant regression equations were found for MEx, but not for nM-EDL and motor-EDL, in the female PD population. However, no significant regression models were found in the male population (Table 3).

**Table 3:** Multiple linear regression analyses in all MOPD participants and stratified by gender.

Independent variable	Total (n = 107)		Male (n = 64)		Female (n = 43)	
	ß ± SE^a^	p-value	ß ± SE^b^	p-value	ß ± SE^b^	p-value
nM-EDL (dependent variable)						
RMET total scores	−0.03 ± 0.02	0.06	−0.03 ± 0.02	0.15	−0.04 ± 0.03	0.18
Positive valence scores	−0.06 ± 0.04	0.13	−0.05 ± 0.04	0.26	−0.06 ± 0.07	0.37
Negative valence scores	−0.05 ± 0.04	0.15	−0.05 ± 0.04	0.30	−0.09 ± 0.07	0.20
Neutral valence scores	−0.04 ± 0.03	0.24	−0.04 ± 0.04	0.32	−0.05 ± 0.06	0.44
motor-EDL (dependent variable)						
RMET total scores	−0.26 ± 0.12	0.04*	−0.15 ± 0.15	0.32	−0.47 ± 0.23	0.05
Positive valence scores	−0.48 ± 0.29	0.10	−0.53 ± 0.34	0.13	−0.27 ± 0.56	0.64
Negative valence scores	−0.62 ± 0.28	0.03*	−0.53 ± 0.35	0.13	−0.89 ± 0.50	0.08
Neutral valence scores	−0.17 ± 0.24	0.49	0.12 ± 0.27	0.65	−0.86 ± 0.47	0.08
MEx (dependent variable)						
RMET total scores	−0.04 ± 0.01	<0.01**	−0.03 ± 0.02	0.05	−0.05 ± 0.02	0.03*
Positive valence scores	−0.04 ± 0.03	0.14	−0.07 ± 0.04	0.07	−0.01 ± 0.05	0.78
Negative valence scores	−0.08 ± 0.03	<0.01**	−0.07 ± 0.04	0.06	−0.11 ± 0.05	0.03*
Neutral valence scores	−0.05 ± 0.02	0.05	−0.02 ± 0.03	0.42	−0.10 ± 0.04	0.02*

Abbreviations: see Tables 1 and 2.

^a^Age, education, gender, disease duration, and LED were adjusted.

^b^Age, education, disease duration, and LED were adjusted.

*P-value < 0.05. **P-value < 0.01.

## Discussion

The primary objective of this study was to investigate affective ToM in patients suffering from YOPD or MOPD. We found that the MOPD patients did not infer emotions as accurately as the YOPD patients or the normal controls did. Further analysis showed that this phenomenon was only found in the female patient population and for specific emotional valences (negative and neutral). Our result was partially in accordance with previous investigations, which recruited patients with YOPD^16^ and MOPD^25,27,29^ to explore their affective ToM ability. Our research echoes previous studies that suggested that PD patients suffered from a selective impairment in facial emotion recognition of negative emotion (i.e., fear and disgust)^32^. The recognition of negative facial emotion expression is encoded through specific neural substrates, such as the basal ganglia, amygdala, anterior cingulate, lateral orbitofrontal cortices, and right inferior prefrontal cortex^32–34^. We further found that patients also have difficulty in inferring another’s neutral emotional state, such as contemplative and reflective state as shown through one’s eyes. To the best of our knowledge, our study is the first to compare the affective ToM ability in YOPD versus MOPD patients directly, and the gender factor was also considered. We demonstrated an impaired affective ToM for neutral valences in female MOPD patients. Our results may resolve the previous controversial findings of the affective ToM in PD patients^9^ by recognizing the heterogeneity in the PD population (i.e., YOPD vs. MOPD and male vs. female). Among healthy people, females tend to be better than males at judging emotions or mental states represented by eye stimuli^35,36^. Nevertheless, our study found that female patients with MOPD were significantly worse than female normal controls and YOPD. This finding may imply that female patients with MOPD have a vulnerable emotional recognition ability; thus, early detection and appropriate treatment are needed for the female MOPD population. Moreover, the underlying mechanism behind the impairment in negative and neutral affective ToM in MOPD patients (especially females) warrants further exploration.

We found that the affective ToM was vulnerable in MOPD, and those patients make up the most common group in PD population^15^. Thus, a deeper understanding about the manifestation of PD as motor and non-motor symptoms should lead to more comprehensive and complete patient care. We further recruited more MOPD patients to determine the relationships between affective ToM and non-motor symptoms. Based on the neuropathology of PD^12^ and reported neuroanatomy responsible for affective ToM^10,11^, we assumed that the affective ToM was related to the motor symptoms. Among MOPD patients, the ability to accurately infer the emotional state of others was positively correlated with patient’s education level and disease duration. However, no correlation was found between dopaminergic treatment and impairment in affective ToM; this finding was consistent with those of previous studies that concluded that LED was not correlated with RMET performance^13,21,25^.

After adjusting for age, education level, disease duration, and levodopa equivalent dose, we found that affective ToM predicted the motor symptoms and motor experiences of daily living. Nevertheless, affective ToM cannot predict non-motor symptoms. This phenomenon was particularly evident in the female MOPD patient population. The current findings confirmed our hypothesis that given the neuropathology of PD^12^ and reported neuroanatomy responsible for affective ToM, the affective ToM and motor symptoms were closely related but not correlated with other non-motor symptoms. Our findings are in line with one recent study^37^, although they are not consistent with Bodden *et al*.^25^. Combining the results in this study and those from Nobis *et al*., we suggest that the correlation between ToM and motor symptoms may be explained by the progression of the disease and pathology. Moreover, PD patients in the early stages of disease progression may have preserved affective ToM^13,21,26^, with difficulties sometimes emerging after 5 years^23^. These findings, along with those of this study, suggest that the PD manifestation may evolve over time, which would stress the importance of continuous monitoring of disease progression in order to supplement therapeutic plans as new needs arise. Furthermore, we found that the RMET total score was negatively correlated with specific motor symptoms, such as bradykinesia, axial symptoms (i.e., gait and postural instability), and impaired speech and facial expression. Our findings partially support those^29^ that have suggested that impaired affective ToM can be associated with the freezing of gait in patients with PD. The correlation is not unreasonable because the processing of affective ToM and axial functions (e.g., gait) may share a common substrate in the ventromedial prefrontal network, according to current understanding. And if we integrate our results with those of De Ferrari *et al*.^29^, it can be suggested that PD patients experiencing bradykinesia or axial motor symptoms are more prone to impaired affective ToM compared to patients with tremor or rigidity motor symptoms. Further studies are needed to confirm that the pathophysiology behind the various motor symptoms in PD has different implications for the patient’s affective ToM abilities. Our study is a cross-sectional study, so we cannot determine the causal relationship between motor symptoms and affective ToM. In light of the rethinking about the PD diagnosis criteria^3^, the diagnosis of PD might shift to the assessment of non-motor symptoms (e.g., dementia) of PD patients. Whether the affective ToM can be used as a biomarker to detect or diagnose PD is a noteworthy issue, and the development of a useful diagnostic tool is therefore needed.

Two small-sample-size studies^21,25^, for example, did not find an association between affective ToM and other non-motor symptoms, such as cognitive function and depressive symptoms. Nevertheless, McKinlay *et al*. suggested that the patient’s cognitive status was a significant predictor of performance in ToM tasks, and the same study found that visuospatial functioning plays an important mediating role in the relationship between executive dysfunction and affective ToM deficits in PD patients^30^. De Ferrari *et al*. found that the affective ToM was positively correlated with the total number of advantageous cards in the Iowa Gambling Task, a well-established task to assess executive function. The ability to infer emotions in others appeared then to be linked to the personal judgment in decision-making and to be an important factor in determining one’s social well-being^29^. The current study found that the MOPD patient’s affective ToM was not related to other non-motor symptoms. Future studies might evaluate the relationship between specific cognitive functions and the various emotional valences.

We recognize the lack of a more fully exploited cognitive function in the participants as one of the study limitations. Future research should investigate cognitive function, especially the patient’s executive function, in a more comprehensive manner to resolve the controversy about the relationships between cognitive function and ToM. Clarifying these relationships may be helpful in improving or retraining ToM when some research has begun to provide insights in this direction^38^. Next, the emotional inference ability was evaluated in the current study solely by observing photographed pairs of eyes, even though in a real social context, the understanding of other people’s emotions may involve both verbal and non-verbal language. Future studies may employ both verbal and non-verbal information in the assessment of affective ToM to provide a more comprehensive context that mimics real-life situations. Third, the current study is a cross-sectional study, and the causal relationship between ToM and clinical characteristics need to be evaluated in a longitudinal study.

In conclusion, the results indicate that MOPD patients experienced deficits in affective ToM for negative- and neutral-specific emotion states, and such deficits are more pronounced in the female patients. In addition, impairment in affective ToM was associated with the development of motor symptoms. Our study emphasizes the heterogeneity of PD patients, and the data suggest that the emotion-decoding ability was preserved in YOPD patients, while the affective ToM was vulnerable in the female MOPD patients. Therefore, the affective ToM in female MOPD patients requires more attention in further investigations. Our findings may aid in the development of educational or medical care programs by showing the need for a more comprehensive therapeutic plan that includes continuous disease progression monitoring and social skills training for PD patients or their caregivers.

## Methods

### Participants

A total of 107 MOPD, 30 YOPD, and 30 healthy participants as NCs were recruited. For the primary study, 60 patients with PD (30 YOPD and 30 MOPD) and 30 NCs were included for the purpose of matching for sex, education level, mental state, disease severity, and levodopa equivalent daily dose. For the second purpose, we further recruited 77 MOPD patients to explore the relationship between affective ToM and PD-related clinical characteristics. All PD patients were diagnosed with PD according to the United Kingdom PD Society Brain Bank clinical diagnostic criteria^39^. Patients were divided into 2 groups according to their age at onset of PD. Those with an onset age less than or equal to 49 years were classified into the YOPD group, and an onset of PD after age 50 and before 70 was classified as MOPD^15^. The NCs were recruited from communities. The exclusion criteria included illiteracy, atypical parkinsonism, a history of psychiatric disorders (e.g., depressive disorders or anxiety disorder), MMSE^40^ score lower than 24, and other neurological or serious systemic disease.

All the participants provided written informed consent prior to enrollment, in accordance with the ethical standards laid down in the 1964 Declaration of Helsinki. All study procedures were approved by the ethical research committee of Kaohsiung Medical University Hospital, and all methods were performed in accordance with the approved guidelines.

### Assessment

#### Reading the mind in the eye test

The RMET is an untimed assessment that comprises 36 black-and-white photographed pairs of eyes that each depict a particular emotion^8^: 8 positive, 12 negative, and 16 neutral^31^ valences, utilizing the same algorithms used by Harkness *et al*.^31^ The participants were asked to pick an adjective (out of 4) that best fit the emotion expressed by the photograph (e.g., which of the following adjectives best describes the eye region shown: excited, relieved, shy, or despondent).

#### Clinical Characteristics

Before taking the RMET, participants underwent a detailed interview to disclose demographic characteristics and clinical characteristics and received a mental state evaluation. In addition, MMSE was used to measure the global mental ability in the study participants. For the patients with PD, we recorded their disease onset age and disease duration and calculated the LED, according to the recommendations of Tomlinson *et al*.^41^. The MDS-UPDRS^42^ and the H&Y staging criteria were used to quantify the motor symptoms and disease severity, respectively. The three parts of the MDS-UPDRS were applied:

Part I: nM-EDL, including non-motor symptoms, such as (1) neuropsychiatric, (2) sleep, and (3) gastrointestinal and autonomic disturbances;

Part II: motor-EDL, composed of 13 patient-based items; and

Part III: Motor Examination (MEx), composed of 18 rater-based objective items.

### Statistical Analysis

All variables were tested for normal distribution using the Kolmogorov-Smirnov test. The study groups were compared using the *t* test and one-way analysis of variance (ANOVA) with the Scheff post hoc test (*P* < 0.05) for parametric variables and the chi-square test, Mann–Whitney U test, and Kruskal–Wallis test for nonparametric variables. Statistical significance was determined when the probability value was less than 0.05. Moreover, multivariate linear regression analyses were performed to explain the relationships between RMET and PD patients’ clinical characteristics. For the multivariate linear regression analysis, RMET total score, positive valence, negative valence and neutral valence scores were considered as independent variables, while dependent variables were nM-EDL, motor-EDL, and MEx. We carried out four multiple linear regression models with adjustment for age, education, gender, duration, and LED in all participants. Furthermore, we stratified the subjects by gender and conducted four multiple linear regression models in males and four in females. All the variables met the assumptions of regression analysis, with the exception of the nM-EDL and MEx. The nM-EDL and MEx were not normally distributed, so the variables were log-transformed, and they had a normal distribution thereafter. Commercially available software (SPSS version 21.0; SPSS Inc., Chicago, IL, USA) was used for the statistical analyses.

## Electronic supplementary material

Supplementary Information
