# Supplementary material for: Emotion-Specific Affective Theory of Mind Impairment in Parkinson’s Disease
Source: Sci Rep. 2018 Oct 30;8:16043. doi: 10.1038/s41598-018-33988-6 (PMC6207749; doi:10.1038/s41598-018-33988-6)
Supplement: Supplementary file 1 — Supplementary Information [file 41598_2018_33988_MOESM1_ESM.docx]

**Emotion-Specific Affective Theory of Mind Impairment in Parkinson’s Disease**

Rwei-Ling Yu, PhD^1,2,3,4^, Po See Chen, MD, PhD^1,2^, Shao-Ching Tu, BS^5^, Wei-Chia Tsao, MD^6^, Chun-Hsiang Tan, MD, PhD^6,7^*

^1^Institute of Behavioral Medicine, College of Medicine, National Cheng Kung University, Tainan, Taiwan

^2^Department of Psychiatry, National Cheng Kung University Hospital, College of Medicine, National Cheng Kung University, Tainan, Taiwan

^3^Institute of Allied Health Sciences, College of Medicine, National Cheng Kung University, Tainan, Taiwan

^4^Department of Neurology, National Taiwan University Hospital, College of Medicine, National Taiwan University, Taipei, Taiwan

^5^School of Medicine, College of Medicine, Kaohsiung Medical University, Kaohsiung, Taiwan

^6^Department of Neurology, Kaohsiung Medical University Hospital, Kaohsiung Medical University, Kaohsiung, Taiwan

^7^Graduate Institute of Clinical Medicine, College of Medicine, Kaohsiung Medical University, Kaohsiung, Taiwan

| **Supplementary Table 1.** The comparison of the correct rate of RMET item in the study group | | | | | | | | | | |
| --- | --- | --- | --- | --- | --- | --- | --- | --- | --- | --- |
|  | NCs (N=30) | YOPD (N=30) | MOPD (N=30) | χ2 test |  |  | NCs (N=30) | YOPD (N=30) | MOPD (N=30) | χ2 test |
| item | n (%) | n (%) | n (%) | p-value |  | item | n (%) | n (%) | n (%) | p-value |
| 1 | 12 (40.0) | 14 (46.7) | 13 (43.3) | 0.87 |  | 19 | 12 (40.0) | 9 (30.0) | 13 (43.3) | 0.54 |
| 2 | 19 (63.3) | 22 (73.3) | 14 (46.7) | 0.10 |  | 20 | 20 (66.7) | 20 (66.7) | 17 (56.7) | 0.65 |
| 3 | 20 (66.7) | 14 (46.7) | 15 (50.0) | 0.25 |  | 21 | 11 (36.7) | 7 (23.3) | 8 (26.7) | 0.50 |
| 4 | 23 (76.7) | 26 (86.7) | 20 (66.7) | 0.19 |  | 22 | 19 (63.3) | 23 (76.7) | 16 (53.3) | 0.17 |
| 5 | 15 (50.0) | 12 (40.0) | 12 (40.0) | 0.67 |  | **23** | **17 (56.7)** | **8 (26.7)** | **5 (16.7)** | **0.003*** |
| 6 | 22 (73.3) | 20 (66.7) | 20 (66.7) | 0.81 |  | 24 | 17 (56.7) | 21 (70.0) | 15 (50.0) | 0.28 |
| 7 | 14 (46.7) | 19 (63.3) | 12 (40.0) | 0.18 |  | 25 | 11 (36.7) | 13 (43.3) | 13 (43.3) | 0.83 |
| 8 | 25 (83.3) | 22 (73.3) | 24 (80.0) | 0.63 |  | 26 | 19 (63.3) | 23 (76.7) | 16 (53.3) | 0.17 |
| 9 | 22 (73.3) | 27 (90.0) | 25 (83.3) | 0.24 |  | 27 | 15 (50.0) | 18 (60.0) | 13 (43.3) | 0.43 |
| 10 | 14 (46.7) | 15 (50.0) | 15 (50.0) | 0.96 |  | 28 | 13 (43.3) | 16 (53.3) | 12 (40.0) | 0.56 |
| 11 | 16 (53.3) | 15 (50.0) | 11 (36.7) | 0.39 |  | **29** | **22 (73.3)** | **27 (90.0)** | **17 (56.7)** | **0.01*** |
| **12** | **22 (73.3)** | **23 (76.7)** | **14 (46.7)** | **0.03*** |  | 30 | 13 (43.3) | 14 (46.7) | 14 (46.7) | 0.96 |
| 13 | 25 (83.3) | 26 (86.7) | 23 (76.7) | 0.59 |  | 31 | 19 (63.3) | 20 (66.7) | 15 (50.0) | 0.38 |
| **14** | **25 (83.3)** | **21 (70.0)** | **16 (53.3)** | **0.04*** |  | 32 | 15 (50.0) | 13 (43.3) | 14 (46.7) | 0.87 |
| **15** | **27 (90.0)** | **20 (66.7)** | **17 (56.7)** | **0.01*** |  | 33 | 15 (50.0) | 15 (50.0) | 13 (43.3) | 0.84 |
| 16 | 13 (43.3) | 22 (73.3) | 16 (53.3) | 0.06 |  | 34 | 13 (43.3) | 14 (46.7) | 14 (46.7) | 0.96 |
| 17 | 16 (53.3) | 18 (60.0) | 16 (53.3) | 0.84 |  | 35 | 21 (70.0) | 21 (70.0) | 13 (43.3) | 0.05 |
| **18** | **21 (70.0)** | **24 (80.0)** | **12 (40.0)** | **0.004*** |  | 36 | 19 (63.3) | 16 (53.3) | 16 (53.3) | 0.67 |
| RMET, Reading the mind in the eyes test  * p<0.05 | | | | | | | | | | |

(O): correct answer; (X): incorrect answer.

**Supplementary Figure 1.** The histogram of each options of item 12 (Neutral).

(O): correct answer; (X): incorrect answer.

**Supplementary Figure 2.** The histogram of each options of item 15 (Neutral).

(O): correct answer; (X): incorrect answer.

**Supplementary Figure 3.** The histogram of each options of item 18 (Neutral).

(O): correct answer; (X): incorrect answer.

**Supplementary Figure 4.** The histogram of each options of item 29 (Neutral).

(O): correct answer; (X): incorrect answer.

**Supplementary Figure 5.** The histogram of each options of item 14 (Negative).

(O): correct answer; (X): incorrect answer.

**Supplementary Figure 6.** The histogram of each options of item 23 (Negative).

| **Supplementary Table 2.** The comparison of the correct rate of RMET item in the **female** group | | | | | | | | | | |
| --- | --- | --- | --- | --- | --- | --- | --- | --- | --- | --- |
|  | NCs (N=19) | YOPD (N=13) | MOPD (N=14) | χ2 test |  |  | NCs (N=19) | YOPD (N=13) | MOPD (N=14) | χ2 test |
| item | n (%) | n (%) | n (%) | p-value |  | item | n (%) | n (%) | n (%) | p-value |
| 1 | 10 (52.6) | 7 (53.9) | 7 (50.0) | 0.98 |  | 19 | 7 (36.8) | 5 (38.5) | 4 (28.6) | 0.86^a^ |
| 2 | 12 (63.2) | 11 (84.6) | 8 (57.1) | 0.32^a^ |  | 20 | 12 (63.2) | 8 (61.5) | 8 (57.1) | 0.94 |
| 3 | 13 (68.4) | 5 (38.5) | 7 (50.0) | 0.23 |  | **21** | **9 (47.4)** | **1 (7.7)** | **2 (14.3)** | **0.04^a^** |
| 4 | 16 (84.2) | 11 (84.6) | 9 (64.3) | 0.43^a^ |  | 22 | 13 (68.4) | 12 (92.3) | 8 (57.1) | 0.14^a^ |
| 5 | 11 (57.9) | 6 (46.2) | 3 (21.4) | 0.11 |  | 23 | 12 (63.2) | 6 (46.2) | 3 (21.4) | 0.06 |
| 6 | 15 (79.0) | 9 (69.2) | 8 (57.1) | 0.47^a^ |  | 24 | 12 (63.2) | 10 (76.9) | 9 (64.3) | 0.73^a^ |
| 7 | 10 (52.6) | 7 (53.9) | 5 (35.7) | 0.55 |  | 25 | 8 (42.1) | 9 (69.2) | 5 (35.7) | 0.18 |
| 8 | 16 (84.2) | 11 (84.6) | 10 (71.4) | 0.64^a^ |  | **26** | **10 (52.6)** | **12 (92.3)** | **8 (57.1)** | **0.046^a^** |
| 9 | 17 (89.5) | 13 (100) | 11 (78.6) | 0.25^a^ |  | 27 | 9 (47.4) | 8 (61.5) | 6 (42.9) | 0.60 |
| 10 | 7 (36.8) | 7 (53.9) | 7 (50.0) | 0.59 |  | 28 | 8 (42.1) | 9 (69.2) | 6 (42.9) | 0.26 |
| 11 | 12 (63.2) | 6 (46.2) | 5 (35.7) | 0.28 |  | **29** | **14 (73.7)** | **12 (92.3)** | **7 (50.0)** | **0.046^a^** |
| 12 | 13 (68.4) | 11 (84.6) | 6 (42.9) | 0.08^a^ |  | 30 | 9 (47.4) | 7 (53.9) | 8 (57.1) | 0.85 |
| 13 | 16 (84.2) | 11 (84.6) | 12 (85.7) | 1.00^a^ |  | 31 | 13 (68.4) | 8 (61.5) | 5 (35.7) | 0.16 |
| **14** | **17 (89.5)** | **10 (76.9)** | **5 (35.7)** | **0.01^a^** |  | 32 | 9 (47.4) | 6 (46.2) | 3 (21.4) | 0.27 |
| **15** | **18 (94.7)** | **10 (76.9)** | **8 (57.1)** | **0.03^a^** |  | 33 | 10 (52.6) | 5 (38.5) | 6 (42.9) | 0.71 |
| 16 | 8 (42.1) | 9 (69.2) | 8 (57.1) | 0.31 |  | 34 | 11 (57.9) | 8 (61.5) | 6 (42.9) | 0.57 |
| 17 | 12 (63.2) | 8 (61.5) | 7 (50.0) | 0.73 |  | 35 | 12 (63.2) | 8 (61.5) | 4 (28.6) | 0.11 |
| 18 | 14 (73.7) | 11 (84.6) | 6 (42.9) | 0.07^a^ |  | 36 | 13 (68.4) | 6 (46.2) | 8 (57.1) | 0.45 |
| RMET, Reading the mind in the eyes test  ^a^ Fisher’s exact test | | | | | | | | | | |

**Supplementary Figure 7.** The histogram of each options of item 15 (Neutral).

**Supplementary Figure 8.** The histogram of each options of item 29 (Neutral).

**Supplementary Figure 9.** The histogram of each options of item 14 (Negative).

**Supplementary Figure 10.** The histogram of each options of item 26 (Negative).

**Supplementary Figure 11.** The histogram of each options of item 21 (Positive).
